# Construct validation of a complete postpartum health and well-being patient reported outcome measure: prospective cohort study

**DOI:** 10.1016/j.xagr.2025.100440

**Published:** 2025-01-21

**Authors:** Laura J. O'Byrne, Gillian M. Maher, Jill M. Mitchell, Ali S Khashan, Richard M. Greene, John P. Browne, Fergus P. McCarthy

**Affiliations:** 1 National Perinatal Epidemiological Centre, University College Cork, Ireland (Byrne and Greene); 2 Department of Obstetrics and Gynaecology, Cork University Maternity Hospital, Ireland (Byrne, Mitchell and McCarthy); 3 INFANT Research Centre, University College Cork, Ireland (Byrne, Maher, Khashan and McCarthy); 4 School of Public Health, University College Cork, Ireland (Maher, Khashan and Browne)

**Keywords:** Childbirth, Maternity PROM, Obstetric care, Patient Reported outcome measures PROM

## Abstract

**Background:**

Despite a focus on patient-reported outcome measures (PROM) in maternity care, a standardized tool is lacking. Current existing measures often focus on a single dimension of postpartum health.

**Objective:**

This study evaluated the construct validity of using a suite of PROMs based on the top psychometrically validated tools available. They were combined to achieve coverage of all important aspects of postpartum well-being outlined by the International Consortium of Health Outcomes (ICHOM).

**Methods:**

Recruitment took place in a tertiary university maternity hospital between April 3^rd^ 2023, and October 28^th^ 2023, with final responses collected in January 2024. Postnatal women were recruited before hospital discharge and consented to completing the PROM tool which consisted of the Postpartum Quality of Life (PQoL) tool, the International Consultation on Incontinence Questionnaire-Urinary Incontinence Short Form (ICIQ-UI SF) and 2 additional questions on pelvic pain with sexual intercourse. The PROM was administered at T1=first week postpartum, T2=6 weeks and T3=12 weeks postpartum. We evaluated the construct validity of these tools through hypothesis testing, proposing that: (1) the instrument should differentiate between groups with and without morbidity, (2) the instrument should differentiate between groups based on delivery type, and (3) should detect change over the postpartum period. Statistical analyses, including chi-square tests, repeated measures ANOVA, and independent t-tests, were used for data analysis.

**Results:**

534 women were recruited, with an average age of 32 years (±5.0), 90.6% (n=484) had term deliveries, 59% (n=316) were multiparous, 40% (n=216) had spontaneous vaginal deliveries (SVD), 12% (n=63) had operative vaginal deliveries and 47.7% (n= 255) had caesarean sections. Examining the tools’ ability to detect changes based on morbidity found no significant differences in PQoL, ICIQ-UI SF or pelvic pain scores between groups with and without maternal morbidity. There were also no differences found in the scores of mothers who had babies admitted to the Neonatal Unit (NNU). Examining score differences based on delivery type, found no variations in total PQoL scores across all timepoints. There were no score differences at other time points in the ICIQ-UI SF or pelvic pain question scores. The PQoL, ICIQ-UI SF and the pelvic pain with sexual intercourse questions had statistically significant difference in their overall scores over the 3 timepoints of the study. The PQoL scores were T1: 128 [±9.67], T2: 125 [±8.47], and T3: 126 [±8.51] *P=*.002. The ICIQ-UI SF had a median score and interquartile ranges of T1: 7.7 (IQR=6), T2: 9 (IQR=7), and T3: 9 (IQR=7), *P=<*.001. The pelvic pain with intercourse questions median score was T1: 6 (IQR=2), T2: 5.5 (IQR=2) and T3: 4 (IQR=2), *P=.*4957.

**Conclusion:**

While this suite of PROMs demonstrated sensitivity in detecting changes in postpartum well-being over time, it did not consistently discern differences based on morbidity (maternal or neonatal) or on delivery type. These findings suggest the need for a more clinically relevant postpartum PROM.

**AJOG Global Reports at a Glance**

**Why was this study conducted?**

To address the notable deficiency in patient-reported outcome measures (PROM) for postnatal care, a composite of top psychometrically evaluated tools was developed. This study assessed the construct validity in relation to postpartum health and well-being by hypothesis testing between clinically relevant subgroups and timepoints.

**Key findings**

No significant differences were detected in postpartum wellbeing between groups with or without maternal or neonatal morbidity. Specific individual morbidities such as admission to the High Dependency Unit (HDU) were initially linked to lower postpartum quality of life scores. However, these variances did not endure across subsequent time intervals, underscoring the intricate interplay of factors affecting postpartum experiences. Differences in scores were detected over 3 time points but their clinical significance is not apparent.

**What does this add to what is known?**

This study contributes to filling the gap in understanding the utilization of PROMs within the maternity system. Despite the availability of top-tier tools, there remains a deficiency in their capacity to differentiate between significant clinically relevant subgroups within the postpartum population.

## Introduction

Patient-reported outcome measures (PROMs) are essential tools for evaluating how healthcare interventions affect an individual's quality of life. They allow nuanced evaluations of different healthcare approaches and interventions, highlighting differences across various demographic and clinical cohorts as well as specific areas that require improvement. Despite the growing focus on PROM in maternity care research over the past decade, there remains a lack of consensus on a standardized PROM tool for this specific healthcare field.^1^, ^2^, ^3^, ^4^ In 2022, our team conducted a systematic review that highlighted the absence of a comprehensive measure available as a postpartum PROM.^3^ This review assessed the psychometric properties of all currently employed PROMs in use for postpartum women, evaluating coverage of the postpartum domains outlined by the International Consortium for Health Outcomes Measurement (ICHOM).^4^ Existing PROMs designed for postpartum women lack comprehensive coverage of postpartum health-related quality of life, frequently concentrating on singular dimensions of postpartum health and well-being, e.g., mental health. This study endeavors to address the deficiencies in this area by constructing a PROM from psychometrically ranking tools that examine all dimensions of postpartum well-being.

The ICHOM framework outlines 6 essential areas for postpartum women: health-related quality of life (HRQoL), mental well-being, transitioning into new roles, breastfeeding, sexual function, and urinary continence. Results of the systematic review^3^ identified the Postpartum Quality of Life (PQoL)^5^ tool as a thorough evaluation of crucial domains like HRQoL, mental health, role transition, and breastfeeding, supported by robust validity, reliability, and evidence. However, it does not encompass assessment of sexual dysfunction and urinary incontinence. To address these gaps, we combined PQoL with International Consultation on Incontinence Questionnaire—Urinary Incontinence Short Form^6^ (ICUI-SF), and included bespoke sexual health inquiries. This enhances the tool's coverage of postpartum health and well-being domains.

This study investigates the construct validity of integrating these top-rated tools to ensure comprehensive domain coverage. Hypotheses for construct validation are formulated concerning the score relationships among distinct patient subgroups with known clinical differences.^7^ It is established that morbidity during childbirth significantly impacts women's psychological and physical well-being, potentially leading to substantial consequences for the dynamics of the family unit as a whole.^8^^,^^9^ The first known sub groups hypothesis to be tested in this work were between morbidity and no morbidity. The second hypothesis was that the tool should be able to differentiate based on delivery type as women with different delivery types have notable differences in their quality of life after childbirth.^10^, ^11^, ^12^ Additionally the tool should be able to discern changes in well-being from the immediate postnatal period to 12 weeks postnatally.

There for our 3 clinically relevant hypotheses for this comprehensive PROM were.

1) ability to discriminate between morbidity groups.
2) ability to discriminate between delivery groups.
3) ability to detect change over the 3 timepoints.

## Materials and methods

### Recruitment

Recruitment was undertaken in a single centre tertiary university maternity hospital with approximately 8000 births per annum, between April 3^rd^ 2023 and October 28^th^ 2023, with final 12-week feedback collected January 2024. In our clinical setting, all women are invited to attend midwife-led antenatal preparation classes, and a postnatal birth reflections clinic is also available for those who wish to participate. The eligibility criteria for recruitment were all inpatients ≥18 years of age on the postnatal wards and post-delivery of >500g baby. Exclusion criteria were those <18 years old, no adequate understanding of spoken or written English and mothers who have experienced a perinatal death. Women were approached by a clinician recruiter, given Patient Information Leaflets (PIL) with a QR code link to the first survey. They consented to demographic details and details regarding delivery being extracted from the electronic health record (EHR), and to submitting their survey responses using the Qualtrics XMTM platform. The data from the survey and the EHR were linked using a unique patient number. This approach was previously examined in a feasibility study in this population with good recruitment and response rates.^13^

### Materials

Demographic information: maternal age, education, parity, and clinical questions regarding the type of delivery and delivery complication were initially collected through the online questionnaire [Appendix]. The combined PROM (generated from the systematic review examining the top psychometrically evaluated tools)^3^ included;PQoL^5^, which consisted of 40 items measured on a 5-point Likert scale. The PQoL encompassed subdomains of childcare (items 1–8), physical function (items 9–20), psychological function (items 21–28), and social support (items 29–40). Response options for these items ranged from “not at all” to “extremely” for intensity, “never” to “always” for frequency, and “very dissatisfied” to “very satisfied” for evaluation. Each PQoL domain was scored by summing the ratings of its respective items. The ICIQ-UI SF^6^ has 3 items which are summed to produce a total score. Higher scores indicate a worse outcome. Two questions pertaining to sexual health were generated by the research team (which includes clinician and psychometrics experts) as this domain had no psychometrically assessed measure found. The first question was as follows: “If you are currently sexually active, do you now experience pain with intercourse”? Responses were measured on a 5-point Likert scale ranging from “None at all” to “A great deal,” with higher scores indicating a worse outcome. The second question was to ascertain if this was a long-standing issue or new post-delivery: “If so, was this something that you had experienced before”? Response options yes/no.[appendix]

## Analysis

### Sample size

Sample size calculations were based on previous feasibility study results^13^ conducted in our population. To observe a difference in PQoL score of 2 (which is less than half a standard deviation in the mean score results) between those who did and did not experience maternal morbidity, a total sample size of 183 was needed to achieve a 5% significance level and 80% power. A total sample size of 250 was required to observe a difference in PQoL score of 2 between delivery types with a 5% significance level and 80% power. Therefore, our objective was to recruit enough women to achieve respondent numbers exceeding 250 at timepoint 1 to account for loss to follow-up, this was estimated to be ∼500 patients.

### Statistical analysis

There were 3 hypotheses; This tool should be able to

1) discriminate between morbidity groups (maternal and neonatal vs no morbidity).
2) discriminate between delivery groups (spontaneous vaginal delivery, operative vaginal delivery, and caesarean section).
3) detect change over the 3 timepoints up to 12 weeks postpartum.

Descriptive statistics were conducted using IBM SPSS. Chi square analysis was used to determine differences between patient characteristics including participants’ age, parity, and delivery type across timepoints. Results of the PQoL scores across the 3 postpartum timepoints were reported as mean score and standard deviation. Mean imputation was used for missing items.^14^ This method entails calculating the average score for the specific subdomain and using it to fill in any missing items within that subset. Specifically, mean imputation was computed based on each participant's subdomain mean score, and this value was used to replace any missing item responses within that subdomain. Results of the ICIQ-UI SF and Pelvic pain with intercourse questions were reported as median score and interquartile range as the data was not normally distributed. Participants who reported no incontinence or who were not sexually active were removed from analysis (absence of incontinence or sexual activity). Repeated measures ANOVA and Friedmann testing were used to identify changes over time points in parametric and nonparametric data respectively. Independent t-tests were conducted on the PQoL data to compare differences in scores between those experiencing a maternal morbidity or not and by neonatal admission to NNU (yes/no). Mann Whitney U testing was performed on the non-parametric ICIQ-UI SF and pain with sexual intercourse question results between those experiencing a maternal morbidity or not and by neonatal admission to NNU. When examining results by delivery type or morbidity type ANOVA was used for normally distributed results and Kruskal Wallis for nonnormally distributed results. Sensitivity analysis was repeated excluding babies who had spent less than 24hrs in NNU. Stata sampsi was used to conduct statistical power analysis.

## Results

Recruitment was performed by a single clinician recruiter; 537 patients were approached, and 534 women consented to participation. The demographic variables are presented in Table 1. The average age of participants was 32 years (±5.0), 44% (n=233) of women had a normal BMI (18.5–25 kg\m^2^). Most women (90.6% (n=484)) experienced term deliveries. There were 59% (n=316) multiparous women, 40% (n=216) had spontaneous vaginal deliveries (SVD), 12% (n=63) had operative vaginal deliveries and 47.7% (n= 255) had caesarean sections (CS). This was subsequently broken down into subgroups of ventouse/kiwi delivery: 8.8% (n=47) and forceps delivery: 3% (n=16) as well as CS being broken down into elective (n=131) 24.5% and emergency (n=124) 23%. An emergency CS being any CS other than an electively pre planned 1.

**Table 1:** Demographics of recruited women from electronic health record and self-reported demographics over the 3 time points

	All recruited n=534 (EHR)	First time point n=334 (self-reported)	Second time point n= 283 (self-reported)	Third time point n= 240 (self-reported)
	n (%)	n (%)	n (%)	n (%)
**Age**		n=334	n=283	n=240
18–24	35 (6.6%)	18 (5%)	14 (4.9%)	10 (4.2%)
25–34	285 (53%)	187 (56%)	144 (50.9%)	120 (50%)
35–44	213 (40%)	129 (38.6%)	125 (44.2%)	110 (45.8%)
>44	1	0	0	0
**Parity**		n=330	n=281	n=236
Primiparous	218 (41%)	153 (46.4%)	153 (54.4%)	107 (45.3%)
Multiparous	316 (59%)	177 (53.6%)	128 (45.6%)	129 (54.7%)
**Education**		n=330	n=281	n=236
Primary		3 (0.9%)	1 (0.4%)	1 (0.4%)
Secondary		62 (18.8%)	46 (16.4%)	44 (18.6%)
University		265 (80.3%)	234 (83.3%)	191 (80.9%)
**Ethnicity**		n=330	n=281	n=236
Irish		258 (78.2%)	217 (77.2%)	182 (77.1%)
European		35 (10.6%)	24 (8.5%)	29 (12.3%)
Asian		22 (6.7%)	21 (7.5%)	14 (5.9%)
African		3 (0.9%)	3 (1.1%)	1 (0.4%)
Other		12 (3.65)	16 (5.7%)	10 (4.2%)
**Delivery**		n=328	n=278	n=235
SVD	216 (40%)	135 (41.2%)	102 (36.7%)	86 (36.6%)
OVD	63 (12%)			
Kiwi Ventouse	47 (8.8%)	30 (9.1%)	24 (8.6%)	21 (8.9%)
Forceps	16 (3%)	9 (2.7%)	10 (3.6%)	6 (2.6%)
C section	255 (47.7%)			
Emergency CS	124 (23%)	75 (22.95%)	69 (24.8%)	50 (21.3%)
Elective CS	131(24.5%)	77 (23.5%)	73 (26.3%)	72 (30.6%)
**Complications**		n=328	n=278	n=235
OASI	8 (1.4%)	9 (2.7%)	10 (3.6%)	8 (3.4%)
PPH	157(29%)	3 (9.1%)	35 (12.5%)	25 (10.6%)
HDU	5 (0.9%)	7 (2.1%)	6 (2.2%)	4 (1.7%)
NNU	51 (10%)	49 (14.9%)	54 (19.4%)	39 (16.6%)
Other*		27 (8.2%)	21 (7.6%%)	10 (4.3%)
**BMI**				
<18.5	3 (0.)			
18.5–25	233 (44%)			
25–30	178 (33%)			
30–40	101 (19%)			
>40	19 (4%)			
**Delivery Gestation**				
<28weeks	2 (0.4%)			
Preterm	48(9%)			
Term	484 (90.6%)			

Recruitment rate was high with only 3 women approached not participating. Response rates at T1 were 63% (n=334), T2 53% (n=283) and T3 45% (n=240). Delivery types were representative of the general hospitals delivery demographics in CUMH with ∼14% instrumental deliveries and a rate of ∼42% for Caesarean section at that time [e Table 1 in appendix]. The demographics of respondents did not change significantly over the 3 timepoints.

### Differences in score by morbidity

#### Maternal morbidity

Women who suffered no maternal morbidity were compared to those who had reported morbidity over 3 timepoints (Table 2). The PQoL score at T1 for those suffering a maternal morbidity did not statistically differ to those without (126.4 (±8.9) n= 234 vs 128.3(±9.9) n=52, *P=*.218). The PQoL has no statistically significant difference across the other 2 timepoints T2 *P=*.517 and T3 *P=*.381. When examining the subdomains of the PQoL, there was no difference between the scores found in the childcare, physical or psychological domains but there was a statistically significant difference in the social domain between the 2 groups at T1, no morbidity vs morbidity (43.8(±5.0) n=233 vs 41.4(±6.1) n=52, *P*=.010). This difference was not seen at T2 *P=*.528 or T3 *P=*.364. The ICIQ-UI SF initially had a difference in scores at T1 7(3.5) n=124 vs 10(14) n=23, p 0.012 between the morbidity and no morbidity groups but this was not found at other timepoints, T2 *P=*.196 and T3 *P=*.096. The pelvic pain with intercourse questions did not suggest statistically significant differences between morbidity and no morbidity over the 3 timepoints.

**Table 2:** Comparing mean PQoL, ICIQ-UI SF and pelvic pain with intercourse scores by maternal and neonatal morbidity at 3 timepoints

	*Maternal Morbidity*			*Neonatal Morbidity*		
Tool Timepoint	No Morbidity Mean (±SD) n=	Morbidity Mean (±SD) n=	Sig.	No NNU Mean (±SD) n=	NNU Mean (±SD) n=	Sig.
PQoL						
T1	128.3 (±9.9) n=234	126.4 (±8.9) n=52	0.218	128.1 (±9.8) n=246	127.2 (±9.2) n=40	0.622
T2	128.3 (±9.9) n=234	124.6 (±8.5) n=54	0.517	125.6 (±7.9) n=203	124.0 (±7.9) n=47	0.247
T3	126.3 (±8.3) n=171	125.0 (±9.1) n=46	0.381	126.3 (±8.6) n=181	124.8 (±7.9) n=36	0.318
PQoL Domain Childcare						
T1	25.0 (±4.3) n=234	25.1 (±3.9) n=52	0.944	25.0 (±4.2) n=246	24.9 (±3.8) n=40	0.907
T2	24.2 (±4.1) n=196	24.3 (±4.7) n=54	0.827	24.4 (±3.9) n=203	23.6 (±3.9) n=47	0.238
T3	24.6 (±4.5) n=171	24.0 (±3.4) n=46	0.419	24.5 (±4.3) n=181	24.3 (±4.0) n=36	0.721
PQoL Domain Physical						
T1	29.7 (±3.7) n=234	30.7 (±3.2) n=52	0.080	29.9 (±3.7) n=246	30.3 (±3.7) n=39	0.507
T2	29.5 (±3.9) n=196	29.2 (±3.1) n=54	0.554	29.5 (±3.9) n=203	29.2 (±3.2) n=47	0.534
T3	30.4 (±3.6) n=171	31.0 (±3.6) n=46	0.277	30.5 (±3.6) n=181	30.8 (±2.8) n=36	0.570
PQoL Domain Psych						
T1	30.1 (±2.1) n=233	29.9 (±2.2) n=51	0.674	30.1 (±2.1) n=245	29.9 (±2.3) n=39	0.741
T2	30.1 (±2.2) n=196	30 (±2.1) n=54	0.798	30.1 (±2.2) n=203	29.9 (±2.2) n=47	0.453
T3	30.2 (±2.3) n=171	29.8 (±2.4) n=46	0.329	30.1 (±2.4) n=181	30.2 (±2.1) n=36	0.962
PQoL Domain Social						
T1	43.8 (±5.0) n=233	41.4 (±6.1) n=52	**0.010**	43.4 (±5.4) n=245	42.8 (±4.5) n=40	0.521
T2	41.6 (±5.6) n=196	41.1 (±6.1) n=54	0.528	41.5 (±5.7) n=203	41.4 (±5.8) n=47	0.887
T3	41.1 (±6.1) n=171	40.2 (±5.8) n=46	0.364	41.2 (±6.1) n=181	39.6 (±6.0) n=36	0.149
ICIQ-UI SF	Median (IQR)	Median (IQR)	Sig.	Median (IQR)	Median (IQR)	Sig.
T1	7 (3.5) n=124	10 (14) n=23	**0.012**	7.5 (4.3) n=126	8 (3.5) n=21	0.562
T2	9 (5) n=94	11.5 (7) n=26	0.196	9 (5) n=94	8.5 (5) n=26	0.401
T3	8 (4) n=82	11 (8) n=23	0.096	9 (4) n=89	8.5 (5) n=16	0.896
Pelvic pain with intercourse	Median (IQR)	Median (IQR)	Sig.	Median (IQR)	Median (IQR)	Sig.
T1	6 (4) n=57	4 (4) n=15	.722	6 (4) n=67	6 (4) n=5	0.329
T2	5 (4) n=66	6 (4) n=14	.649	6 (4) n=68	5 (4) n=12	0.066
T3	4.5 (4) n=118	3 (4) n=25	.816	4 (4) n=122	6 (4) n=21	0.520

T1 – T1 – timepoint 1 (within 1 week of delivery). T2 – timepoint 2 (6 weeks post-delivery). T3 – timepoint 3 (12 weeks post-delivery.

#### Neonatal unit admission (NNU)

We compared the scores in relation to NNU admission (Table 2). The total PQoL scores between these groups at T1 were (128.1(±9.8) n=246 vs 127.2(±9.2) n=40, *P=*.622), T2 (125.6(±7.9) n=203 vs 124.0(±7.9) n=47, *P=*.247) and T3 (126.3(±8.6) n=181 vs 124.8(±7.9) n=36 *P=*.318). No statistically significant differences existed between these groups (Table 2).

Similarly, for the ICIQ-UI SF and the pelvic pain questions, no significant difference existed at any time point in relation to NNU admission. Results remained unchanged when we conducted sensitivity analysis excluding babies with <24hr admission to NICU (e table 2).

#### Maternal morbidity type

We repeated the analysis according to the following morbidity types: postpartum haemorrhage (PPH), high dependency unit (HDU) admission, obstetric anal sphincter injury (OASIS) and other reported outcome morbidity, e.g., epidural failure and manual removal of placenta. (Table 3) There were statistically significant differences in the total PQoL scores at T1 *P=*.001 with high dependency unit admission scoring lowest 119.1 (±11.4) and OASIS scoring highest 132.5(±8.9). The individual sub domains that had differences in scores at T1 were the childcare domain *P=*.040 and the social domain *P=*.039. In the childcare domain the PPH group scored lowest (23.9(±3.3)), and the ‘other’ morbidity scored highest (27.1(±3.7)). This difference in scores was not seen at T2 *P=*.647 or T3 *P=*.322 with the social domain HDU admission scoring lowest: 38.6 (±5.6) and OASIS scoring highest: 45.5(±6.3). The groups did not differ on total PQoL score or in any subdomains at T2 or T3. There were no differenced observed across morbidity groups in the ICIQ-UI SF and the pelvic pain questions.

**Table 3:** Comparing mean PQoL, ICIQ-UI SF and pelvic pain with intercourse scores by maternal morbidity type at 3 timepoints

Tool Subdomain Timepoint	PPH Mean (±SD) n=	HDU admission Mean (±SD) n=	OASIS Mean (±SD) n=	Other Mean (±SD) n=	Sig.
Total PQoL					
1	125.3 (±7.3) n=24	119.1 (±11.4) n=7	132.5 (±8.9) n=8	131.8 (±8.2) n=27	**0.001**
2	124.3 (±9.7) n=30	120.1 (±7.4) n=4	124.2 (±7.6) n=9	126.6 (±7.8) n=17	0.582
3	124.8 (±9.6) n=24	122.2 (±6.0) n=4	123.7 (±9.8) n=7	126.0 (±9.1) n=16	0.870
PQoL Domain Childcare					
1	23.9 (±3.3) n=24	24.7 (±4.8) n=7	26.2 (±5.3) n=8	27.1 (±3.7) n=27	**0.040**
2	24.2 (±3.8) n=30	23.4 (±4.4) n=4	23.9 (±5.4) n=9	25.9 (±6.8) n=17	0.647
3	24.1 (±2.7) n=24	21.4 (±2.1) n=4	23.3 (±5.4) n=7	25.3 (±5) n=16	0.322
PQoL Domain Physical					
1	31.0 (±3.3) n=24	30.9 (±4.1) n=7	30.8 (±3.1) n=8	30.7 (±3.2) n=27	0.994
2	29.8 (±3.4) n=30	28.3 (±3.0) n=4	27.7 (±1.9) n=9	29.6 (±3.8) n=17	0.358
3	21.0 (±3.9) n=24	31.0 (±2.2) n=4	30.3 (±2.7) n=7	30.1 (±2.7) n=16	0.829
PQoL Domain Psych					
1	29.8 (±2.5) n=24	29.0 (±2.0) n=7	30.1 (±2.4) n=8	30.2 (±1.8) n=27	0.630
2	29.9 (±2.3) n=30	29.8 (±2.5) n=4	29.4 (±1.3) n=9	30.0 (±2.6) n=17	0.947
3	29.5 (±2.5) n=24	30.0 (±0) n=4	28.9 (±1.3) n=7	30.9 (±2.7) n=16	0.179
PQoL Domain Social					
1	40.7 (±6.5) n=24	38.6 (±5.6) n=7	45.5 (±6.3) n=8	43.7 (±4.9) n=27	**0.039**
2	40.5 (±5.9) n=30	38.8 (±7.4) n=4	43.2 (±3.3) n=9	41.1 (±6.9) n=17	0.569
3	40.2 (±5.7) n=24	39.8 (±9.2) n=4	41.1 (±6.8) n=7	39.7 (±4.5) n=16	0.955
ICIQ-UI SF	Median (IQR)	Median (IQR)	Median (IQR)	Median (IQR)	
1	10 (3.6) n=12	9.5 (0) n=2	11 (0) n=2	7 (6.3) n=11	0.365
2	12.5 (5) n=18	- (-) n=1	14 (-) n=3	8 (3) n=7	0.131
3	11.5 (6) n=12	9 (-) n=3	6 (0) n=2	7 (9) n=7	0.093
Pelvic pain with intercourse	Median (IQR)	Median (IQR)	Median (IQR)	Median (IQR)	
1	6 (2) n=6	- (-) n=1	- (-) n=1	3 (3) n=10	-
2	6 (4) n=9	- (-) n=1	2 (4) n=3	5 (-) n=2	0.617
3	6 (4) n=11	3 (-) n=3	2 (0) n=4	5 (4) n=9	0.125

T1 – timepoint 1 (within 1 week of delivery). T2 – timepoint 2 (6 weeks post-delivery). T3 – timepoint 3 (12 weeks post-delivery.

### Differences in score by delivery type

Differences in scores by delivery type are seen in Table 4. At T1 the PQoL results across delivery types showed no statistically significant difference, *P=*.934. Spontaneous vaginal delivery (SVD) had the highest total score: 128.5 (±7.6) and operative vaginal delivery with kiwi had the lowest: 127.0 (±17.8), although the difference was minimal. There was no statistical difference found at T2 *P*=.354 or T3 *P=*.369. There were no subdomain differences detected between delivery types in childcare, physical, psychological and the social domain over the 3 timepoints.

**Table 4:** Comparing mean scores for PQoL, ICIQ-UI SF and pelvic pain with intercourse tools by mode of delivery type at 3 timepoints

Tool Subdomain Timepoint	Spontaneous vaginal delivery Mean (±SD) n=	Operative vaginal delivery Kiwi Mean (±SD) n=	Operative vaginal delivery Forceps Mean (±SD) n=	Emergency C Section Mean (±SD) n=	Elective C Section Mean (±SD) n=	Sig.
Total PQoL						
1	128.5 (±7.6) n=116	127.0 (±17.8) n=25	128.0 (±5.9) n=7	127.8 (±9.3) n=65	127.6 (±9.7) n=71	0.934
2	125.2 (±7.3) n=90	126.3 (±9.5) n=23	119.1 (±6.9) n=7	125.9 (±9.6) n=60	125.1 (±8.6) n=70	0.354
3	126.6 (±7.9) n=79	127.3 (±9.9) n=20	120 (±7.7) n=6	126.4 (±8.9) n=47	125.4 (±8.6) n=65	0.369
PQoL Domain Childcare						
1	25.3 (±4.4) n=116	24.9 (±4.3) n=25	25.7 (±4.5) n=7	24.6 (±4.4) n=65	24.9 (±3.6) n=71	0.937
2	24.3 (±4.4) n=90	24.9 (±4.1) n=23	22.0 (±7.0) n=7	23.9 (±4.7) n=60	24.4 (±3.8) n=70	0.581
3	24.6 (±4.3) n=79	25.3 (±4.1) n=20	22.1 (±3.5) n=6	24.6 (±4.6) n=47	24.3 (±4.6) n=65	0.576
PQoL Domain Physical						
1	29.8 (±3.3) n=116	29.7 (±4.1) n=25	28.4 (±5.7) n=7	30.0 (±3.2) n=65	30.3 (±4.4) n=71	0.343
2	30.1 (±2.2) n=90	28.8 (±2.2) n=23	29.6 (±1.8) n=7	30.4 (±1.8) n=60	30.2 (±2.4) n=70	0.322
3	30.6 (±3.4) n=79	30.4 (±4.5) n=20	30.4 (±3.4) n=6	30.5 (±3.5) n=47	30.4 (±3.5) n=65	0.999
PQoL Domain Psych						
1	30.0 (±2.1) n=116	30.1 (±2.5) n=25	30.4 (±2.1) n=7	39.8 (±2.1) n=65	30.2 (±2.0) n=71	0.871
2	29.1 (±3.6) n=90	29.4 (±4.1) n=23	27.6 (±3.3) n=7	30.1 (±4.2) n=60	29.6 (±3.5) n=70	0.053
3	30.2 (±2.2) n=79	28.8 (±3.1) n=20	29.2 (±1.7) n=6	30.4 (±2.4) n=47	30.4 (±2.2) n=65	0.066
PQoL Domain Social						
1	43.4 (±5.2) n=116	45.3 (±5.0) n=25	43.5 (±4.7) n=7	43.4 (±5.6) n=65	42.6 (±5.4) n=71	0.488
2	41.7 (±5.7) n=90	43.3 (±5.5) n=23	40 (±3.7) n=7	41.4 (±6.3) n=60	40.9 (±5.5) n=70	0.444
3	41.2 (±5.8) n=79	42.8 (±4.7) n=20	38.3 (±5.7) n=6	40.9 (±7.0) n=47	40.3 (±6.0) n=65	0.443
ICIQ-UI SF	Median (IQR)	Median (IQR)	Median (IQR)	Median (IQR)	Median (IQR)	
1	7.7 (5) n=63	8 (4) n=15	7.7 (5) n=4	7.7 (5) n=28	7.7 (5) n=36	0.601
2	9 (5) n=57	12.5 (6) n=16	13.5 (8) n=4	8 (4) n=18	8 (5) n=25	0.146
3	9 (4) n=47	9 (2) n=12	14 (0) n=3	9.5 (4) n=18	8 (6) n=25	0.913
Pelvic pain with intercourse						
1	4 (4) n=27	4 (4) n=4	0 n=0	6 (4) n=15	6 (4) n=25	0.526
2	6 (4) n=29	4 (4) n=8	0 n=0	2 (4) n=16	6 (4) n=27	0.257
3	6 (4) n=58	2 (2) n=14	2 (4) n=3	6 (4) n=26	4 (4) n=42	0.233

T1 – timepoint 1 (within 1 week of delivery). T2 – timepoint 2 (6 weeks post-delivery). T3 – timepoint 3 (12 weeks post-delivery.

The ICIQ-UI SF and Pelvic pain with intercourse when examined by delivery type revealed no statistically significant difference in scores by delivery type across all 3 timepoints.

#### Responsiveness

The mean results of respondents to the MOMs questionnaire are represented in Table 5. The changes in the PQoL score were T1: 128 [±9.67], T2: 125 [±8.47], and T3: 126 [±8.51] *P=*.002. Mean scores for the ICIQ-UI SF were as follows: T1: 7.7 (IQR=6), T2: 9 (IQR=7), and T3: 9 (IQR=7), *P=<*.001. The pelvic pain with intercourse questions median score and IQR were T1: 6 (IQR=2), T2: 5.5 (IQR=2) and T3: 4 (IQR=2), *P=*.4957.

**Table 5:** Total population mean ICIQ-UI SF, pelvic pain with intercourse and PQoL scores over 3 timepoints

ToolSubdomain	T1 Mean (±SD)	T2 Mean (±SD)	T3 Mean (±SD)	Sig
PQoL	127.94 (±9.67) n=286	125.27 (±8.47) n=250	126.06 (±8.51) n=217	0.002
Childcare	25.00 (±4.18) n=286	24.21 (±4.24) n=250	24.49 (±4.29) n=217	
Physical	29.92 (±3.70) n=286	29.47 (±3.77) n=250	30.50 (±3.51) n=217	
Psychological	30.03 (±2.10) n=284	30.07 (±2.19) n=250	30.15 (±2.36) n=217	
Social	43.35 (±5.30) n=285	41.51 (±5.74) n=250	40.92 (±6.07) n=217	
**Tool**	**T1 Median (IQR)**	**T2 Median (IQR)**	**T3 Median (IQR)**	**Sig**
ICIQ-UI SF	7.7 (6) n=147	9 (7) n=120	9 (7) n=105	<0.001
Pelvic pain with intercourse	6 (2) n=72	5.5 (2) n=80	4 (2) n=143	0.4957

## Comment

### Principle findings

The combination of these tools for a complete postpartum PROM could not determine clinically relevant differences in patient groups based on the hypotheses proposed in this work. Examining the postpartum PROMs ability to detect changes based on morbidity, found no significant differences in PQoL, or pelvic pain scores between groups with and without maternal morbidity or among mothers who had babies admitted to NNU, while a difference in ICIQ-UI SF scores seen at T1 only. Examining differences based on delivery type, found no variations in total PQoL ICIQ-UI SF or pelvic pain scores. When subgroup analysis focusing on the type of maternal morbidities was conducted, the PQoL revealed statistically significant disparities in total PQoL scores at the first timepoint within 1 week of delivery, specifically, women admitted to the HDU exhibited the lowest scores at 119.1(±11.4), while those with obstetric anal sphincter injury (OASIS) recorded the highest scores at 132.5(±8.9). Additionally, within the childcare domain, individuals in the PPH group scored the lowest at 23.9(±3.3), possibly due to profound fatigue following significant blood loss.^15^ Remarkably, these PQoL score differences were not evident at any other time point. There were no differences observed across morbidity groups in the ICIQ-UI SF and the pelvic pain questions. Despite there being statistically significant differences in the scores over the 3 timepoints (hypothesis 3) in the PQoL and ICIQ-UI SF, their clinical relevance is questionable. Looking at the minimal clinically important difference (defined as the smallest change on a measure that is reliably associated with a meaningful change in a patient's clinical status, function, or quality of life)^16^ which is 0.5 Standard Deviation of the HRQoL mean, neither reach this threshold.

### Results in context to what is known

The findings of the study are consistent with existing knowledge regarding the challenges of effectively utilizing PROMs in the maternity.^1^^,^^4^^,^^17^^,^^18^ Although validated tools exist, their ability to capture meaningful distinctions among diverse subgroups within the postpartum population remains limited.^17^^,^^19^ This highlights the ongoing necessity for refining and validating PROMs to better address the complexities of postpartum outcomes. There is a need for greater focus on centering women's perspectives in instrument development to ensure relevance, comprehensiveness, and clarity in measurement. Prioritizing women's voices in determining what to measure can enhance overall validity, reliability, and real-world usability of PROMs.^17^

While previous studies, such as the ICIQ-UI SF detected variations among women at 6 weeks postpartum based on delivery method, including SVD, instrumental deliveries, and C-sections^6^ few have examined a multi-morbid population to ascertain clinically relevant differences in outcome.

### Clinical implications

Clinicians should acknowledge the current limitations of PROM in fully capturing the nuanced experiences of postpartum women. This underscores the ongoing need for refinement and validation of these tools to ensure they accurately reflect the diverse postpartum experiences.

Regarding clinical sensitivity, it is essential for a postpartum PROM to effectively discern differences within the populations it assesses. However, the PQoL, ICIQ-UI SF, and pelvic pain questions used in our study did not demonstrate this ability. This prompts critical evaluation of their clinical sensitivity and calls for further refinement or exploration of alternative measures that may better capture the complexities of postpartum well-being.

### Research implications

This study underscores the importance of developing postpartum PROM that are capable of discerning differences within the populations they aim to assess. For a PROM to serve as a useful tool in clinical practice, it must be sensitive enough to capture these variations and provide meaningful insights into the diverse needs and experiences of postpartum women. As such, further research is needed to refine and validate PROMs specific to the postpartum period, ensuring they accurately reflect the multifaceted nature of postpartum well-being.

### Strengths

This study built on the background of a feasibility study^13^ examining the methodological approach, statistical analysis and population sample size required for this work. The large sample size predetermined for this study allowed us to make the statistical inferences. The recruitment rate was satisfactory, with a negligible percentage of women declining to participate, indicating strong engagement and willingness among the target population.

### Weaknesses

While the study includes a substantial sample size, its focus on a single tertiary care Center may limit the generalizability of findings to broader populations or settings. Despite high response rates, the possibility of selection bias cannot be entirely ruled out. However, only 3 people approached declined to participate, and the distribution of demographics are similar among those who participated at T1 and T3. In this work we examined postpartum wellbeing up to 12 weeks postpartum, further work examining women's health and wellbeing up to 6 months could offer more insights into long-term wellbeing.

## Conclusion

The study examined the construct validity of a comprehensive postpartum health assessment by combining the PQoL tool with supplementary measures including the ICIQ-UI SF for urinary incontinence and sexual health inquiries. Significant differences in PQoL, ICIQ-UI SF, and pelvic pain scores based on maternal morbidity, NNU admission, or delivery type were not consistently found. Future work should examine the construction of clinically relevant tools in postnatal setting.

## CRediT authorship contribution statement

**Laura J. O'Byrne:** Writing – original draft, Validation, Methodology, Investigation, Formal analysis. **Gillian M. Maher:** Writing – review & editing, Validation, Methodology, Formal analysis, Conceptualization. **Jill M. Mitchell:** Investigation, Formal analysis. **Ali S Khashan:** Writing – review & editing, Supervision, Methodology, Formal analysis, Conceptualization. **Richard M. Greene:** Writing – review & editing, Supervision, Methodology, Conceptualization. **John P. Browne:** Writing – review & editing, Validation, Supervision, Methodology, Conceptualization. **Fergus P. McCarthy:** Writing – review & editing, Supervision, Methodology, Funding acquisition, Formal analysis, Conceptualization.

## Appendix Supplementary materials

Image, application 1

## Footnotes

Supplementary material associated with this article can be found in the online version at doi:10.1016/j.xagr.2025.100440.
