# Supplementary material for: Construct validation of a complete postpartum health and well-being patient reported outcome measure: prospective cohort study
Source: AJOG Glob Rep. 2025 Jan 21;5(1):100440. doi: 10.1016/j.xagr.2025.100440 (PMC11867515; doi:10.1016/j.xagr.2025.100440)
Supplement: Supplementary file 1 [file mmc1.docx]

**eTable 1:** Delivery demographics from recruited cohort and hospital population.

|  | All recruited | First time point  (self-reported) | Second time point  (self-reported) | Third time point  (self-reported) | CUMH November 2022 | | CUMH  December 2023 |
| --- | --- | --- | --- | --- | --- | --- | --- |
| Total | 534 | 334 | 283 | 240 | | 566 | 564 |
| OVD | 63 (12%) | 39 (12%) | 34 12% | 27 (11%) | | 13.4% | 15.4% |
| Kiwi | 47 (8.8%) | 30 (9.1%) | 24 (8.6%) | 21 (8.9%) | | - | - |
| Forceps | 16(3%) | 9 (2.7%) | 10 (3.6%) | 6 (2.6%) | | - | - |
| Rate of CS | 255 (48%) | 77 (46%) | 142 (50%) | 122 (50%) | | 41.7% | 43.2% |
| Emergency | 124 (23%) | 75 (22.9%) | 69 (24.8%) | 50 (21.3%) | | - | - |
| Elective | 131 (24.5%) | 77 (23.5%) | 73 (26.3%) | 72 (30.6%) | | - | - |

Data from the HSE Maternity Patient safety statements – available online https://www.hse.ie/eng/services/list/3/maternity/mpss/2023/november-2023/november-2023-maternity-patient-safety-statements.html

<https://www.hse.ie/eng/services/list/3/maternity/mpss/2023/december-2023/december-2023-maternity-patient-safety-statements.html>

**eTable 2:** Sensitivity analysis examining neonatal admission to NNU for > 24 hrs alongside all NNU admission over any NNU admission period.

|  | ***Neonatal Morbidity >42 hours*** | | | ***Neonatal Morbidity (all admission)*** | | |
| --- | --- | --- | --- | --- | --- | --- |
| **Tool**  **Timepoint** | **No NNU**  **Mean(±SD)**  **n=** | **NNU >24hrs**  **Mean(±SD)**  **n=** | **Sig.** | **No NNU**  **Mean(±SD)**  **n=** | **NNU**  **Mean(±SD)**  **n=** | **Sig.** |
| PQoL |  |  |  |  |  |  |
| T1 | 128.3(±9.9)  n=234 | 127.4 (±9.5)  n=37 | 0.701 | 128.1(±9.8)  n=246 | 127.2(±9.2)  n=40 | 0.622 |
| T2 | 125.6(±7.9)  n=203 | 123.9 (±8.0)  n=45 | 0.250 | 125.6(±7.9)  n=203 | 124.0(±7.9)  n=47 | 0.247 |
| T3 | 126.3(±8.6)  n=181 | 124.8(±7.9)  n=36 | 0.318 | 126.3(±8.6)  n=181 | 124.8(±7.9)  n=36 | 0.318 |

**Questionnaire**

**MOMs Study: Maternal Outcome Measures for postpartum women.**

**Demographic Data Questions:**

**What age were you at the time of your delivery?**

[*]

**What is the highest education level you received?**

[*] Primary

[*] Secondary

[*] University Post Graduate degree

**What is your ethnicity?**

[*] Irish

[*] European

[*] Asian

[*] African

**Have you given birth before having this baby?**

[*] Y/N

**If yes, how many babies have you had before?**

[*]

**Did you have a singleton or multiple birth?**

[*] 1 Baby

[*] Twins

[*] Triplets

[*] Other

**What way were your baby(ies) delivered?**

[*] Vaginal Delivery

[*] Vaginal Delivery with Suction (Kiwi or Ventouse)

[*] Vaginal Delivery with Forceps

[*] Emergency C-Section

[*] Elective C-Section

[*] Other

**Did you have any delivery complications? Y/N**

[*] Haemorrhage (Large blood loss at the time of delivery)

[*] Admission to High Dependency Unit (HDU)

[*] 3^rd^ or 4^th^ degree tear

[*] Other

**Did your baby(ies) spend time in the SCBU/NICU? Y/N**

If yes, how many days [*]

How many days did you stay in hospital after delivery?

[*]

**Postpartum Women’s Quality of Life Questionnaire**

**Do you worry that your child will fall sick?**

[*] Never

[*] Rarely

[*] Sometimes

[*] Often

[*] Always

**How satisfied are you with your child’s health?**

[*] Very dissatisfied

[*] Dissatisfied

[*] Neither satisfied nor dissatisfied

[*] Satisfied

[*] Very Satisfied

**Do you worry that your child will have an accident?**

[*] Never

[*] Rarely

[*] Sometimes

[*] Often

[*] Always

**How much do you take pains to prevent an accident to your child?**

[*] Not at all

[*] A little

[*] A moderate amount

[*] Very much

[*] An extreme amount

**Do you worry about the nutrition of your child?**

[*] Never

[*] Rarely

[*] Sometimes

[*] Often

[*] Always

**Do you worry that your child is not smart?**

[*] Never

[*] Rarely

[*] Sometimes

[*] Often

[*] Always

**Do you think that your breast milk is enough for your child?**

[*] Not enough at all

[*] Not enough

[*] Sometimes

[*] Enough

[*] Always enough

**How satisfied are you with current feeding?**

[*] Very dissatisfied

[*] Dissatisfied

[*] Neither satisfied nor dissatisfied

[*] Satisfied

[*] Very Satisfied

**Do you worry about unexpected pregnancy?**

[*] Never

[*] Rarely

[*] Sometimes

[*] Often

[*] Always

**How much are you bothered by contraception?**

[*] Not at all

[*] Slightly

[*] Moderately

[*] Very Extremely

**How satisfied are you with your sleep?**

[*] Very dissatisfied

[*] Dissatisfied

[*] Neither satisfied nor dissatisfied

[*] Satisfied

[*] Very Satisfied

**Do you have enough time to rest?**

[*] Never

[*] Rarely

[*] Sometimes

[*] Often

[*] Always

**How easily do you get tired?**

[*] Not at all

[*] Slightly

[*] Moderately

[*] Very Extremely

**How satisfied are you with the energy that you have?**

[*] Very dissatisfied

[*] Dissatisfied

[*] Neither satisfied nor dissatisfied

[*] Satisfied

[*] Very Satisfied

**Does physical pain influence your daily life?**

[*] Never

[*] Rarely

[*] Sometimes

[*] Often

[*] Always

**How much do you think that your physical health has been affected by childbirth?**

[*] Not at all

[*] Slightly

[*] Moderately

[*] Very Extremely

**How much conflict do you feel between childcare and work?**

[*] Not at all

[*] Slightly

[*] Moderately

[*] Very Extremely

**Has your child caused you to be distracted and worried at work?**

[*] Not at all

[*] Slightly

[*] Moderately

[*] Very Extremely

**How satisfied are you with the way your body looks?**

[*] Very dissatisfied

[*] Dissatisfied

[*] Neither satisfied nor dissatisfied

[*] Satisfied

[*] Very Satisfied

**Do you feel blue by your looks?**

[*] Never

[*] Rarely

[*] Sometimes

[*] Often

[*] Always

**How much confidence do you have in caring for your baby well?**

[*] Not at all

[*] A little

[*] A moderate amount

[*] Very much

[*] An extreme amount

**How much childcare skill do you think you have?**

[*] Not at all

[*] A little

[*] A moderate amount

[*] Very much

[*] An extreme amount

**Are you interested in your child?**

[*] Never

[*] Rarely

[*] Sometimes

[*] Often

[*] Always

**Are you willing to look after your child?**

[*] Never

[*] Rarely

[*] Sometimes

[*] Often

[*] Always

**Do you regret having had this child?**

[*] Never

[*] Rarely

[*] Sometimes

[*] Often

[*] Always

**Is caring a baby hard for you?**

[*] Never

[*] Rarely

[*] Sometimes

[*] Often

[*] Always

**Are you happy being a mother?**

[*] Never

[*] Rarely

[*] Sometimes

[*] Often

[*] Always

**How much fun is your life after having this child?**

[*] Not at all

[*] Slightly

[*] Moderately

[*] Very much

[*] Extremely

**Do you have enough contact with the outside world?**

[*] Not at all

[*] Slightly

[*] Moderately

[*] Very much

[*] Extremely

**Do you see enough of your neighbours?**

[*] Not at all

[*] Slightly

[*] Moderately

[*] Very much

[*] Extremely

**What do you think your husband’s attitude is towards you?**

[*] Very bad

[*] Bad

[*] Moderately

[*] Neither bad nor good

[*] Good

[*] Very Good

**How close is the relationship between you and your husband?**

[*] Not at all

[*] Slightly

[*] Moderately

[*] Very Extremely

**How much help do you get caring for your child?**

[*] None at all

[*] A little

[*] A moderate amount

[*] Very much

[*] A great deal

**How much help do you get doing household chores?**

[*] None at all

[*] A little

[*] A moderate amount

[*] Very much

[*] A great deal

**How clean is your house?**

[*] Not at all

[*] Slightly

[*] Moderately

[*] Very Extremely

**How satisfied are you with your housing situation?**

[*] Very dissatisfied

[*] Dissatisfied

[*] Neither satisfied nor dissatisfied

[*] Satisfied

[*] Very Satisfied

**Is the money that yourself can decide how to spend enough?**

[*] Not enough

[*] A little short

[*] Just enough

[*] Enough

[*] Very Enough

**Do you worry about your finances?**

[*] Never

[*] Rarely

[*] Sometimes

[*] Often

[*] Always

**How satisfied are you with your living environment, including pollution, noise, climate and location?**

[*] Very dissatisfied

[*] Dissatisfied

[*] Neither satisfied nor dissatisfied

[*] Satisfied

[*] Very Satisfied

**How satisfied are you with the transportation available to you?**

[*] Very dissatisfied

[*] Dissatisfied

[*] Neither satisfied nor dissatisfied

[*] Satisfied

[*] Very Satisfied

**International Consultation on Incontinence Questionnaire—Urinary Incontinence Short Form**

**1. How often do you leak urine?**

[*] Never

[*] About once a week or less

[*] Two or three times a week

[*] About once a day

[*] Several times a day

[*] All the time

**2. We would like to know how much urine you think leaks. How much urine do you usually leak (whether you wear protection or not)?**

[*] None

[*] A small amount

[*] A moderate amount

[*] A large amount

**3. Overall, how much does leaking urine interfere with your everyday life?**

0, 1, 2,3,4,5,6,7,8,9.10

**When does urine leak?**

[*] Never urine does not leak

[*] Leaks before you can get to the toilet

[*] Leaks when you cough or sneeze

[*] Leaks when you are asleep

[*] Leaks when you are physically active/exercising

[*] Leaks when you have finished urinating and are dressed

[*] Leaks for no obvious reason

[*] Leaks all the time

**Sexual Health Questions:**

**If you are currently sexually active, do you now experience pain with intercourse?**

[*] None at all

[*] A little

[*] A moderate amount

[*] Very much

[*] A great deal

**If so, was this something that you had experienced before?**

[*] Y/N

**Content Validation Questions**

**Do you think this questionnaire captures the most important outcomes that matter or have mattered to you?**

[*] Y/N

**If not, what would you like to add or remove?**

[*] Free Text
